# Isolated nigral degeneration without pathological protein aggregation in autopsied brains with *LRRK2* p.R1441H homozygous and heterozygous mutations

**DOI:** 10.1186/s40478-018-0617-y

**Published:** 2018-10-17

**Authors:** Masashi Takanashi, Manabu Funayama, Eiji Matsuura, Hiroyo Yoshino, Yuanzhe Li, Sho Tsuyama, Hiroshi Takashima, Kenya Nishioka, Nobutaka Hattori

**Affiliations:** 1 Department of Neurology, Juntendo Koshigaya Hospital, 560, Fukuroyama, Koshigaya-city, Saitama, 343-0032 Japan; 2 Research Institute for Diseases of Old Age, Graduate School of Medicine, Juntendo University, 2-1-1 Hongo, Bunkyo, Tokyo, 113-8421 Japan; 3 Laboratory of Genomic Medicine, Center for Genomic and Regenerative Medicine, Graduate School of Medicine, Juntendo University, 2-1-1 Hongo, Bunkyo, Tokyo, 113-8421 Japan; 4 Department of Neurology, Juntendo University School of Medicine, 2-1-1, Hongo, Bunkyo, Tokyo, 113-0033 Japan; 5 Department of Neurology and Geriatrics, Graduate School of Medical and Dental Sciences, Kagoshima University, 8-35-1, Sakuragaoka, Kagoshima-city, Kagoshima, 890-8520 Japan; 6 Department of Human Pathology, Juntendo University School of Medicine, 2-1-1, Hongo, Bunkyo, Tokyo, 113-0033 Japan

**Keywords:** *LRRK2*, P.R1441H, Parkinson’s disease, Pathology, Isolated nigral degeneration, Makurazaki

## Abstract

*Leucine-rich repeat kinase 2* (*LRRK2*) is the most common causative gene for autosomal dominant Parkinson’s disease (PD) and is also known to be a susceptibility gene for sporadic PD. Although clinical symptoms with *LRRK2* mutations are similar to those in sporadic PD, their pathologies are heterogeneous and include nigral degeneration with abnormal inclusions containing alpha-synuclein, tau, TAR DNA-binding protein 43, and ubiquitin, or pure nigral degeneration with no protein aggregation pathologies. We discovered two families harboring heterozygous and homozygous c.4332 G > A; p.R1441H in *LRRK2* with consanguinity, sharing a common founder. They lived in the city of Makurazaki, located in a rural area of the southern region, the Kagoshima prefecture, in Kyushu, Japan. All patients presented late-onset parkinsonism without apparent cognitive decline and demonstrated a good response to levodopa. We obtained three autopsied cases that all presented with isolated nigral degeneration with no alpha-synuclein or other protein inclusions. This is the first report of neuropathological findings in patients with *LRRK2* p.R1441H mutations that includes both homozygous and heterozygous mutations. Our findings in this study suggest that isolated nigral degeneration is the primary pathology in patients with *LRRK2* p.R1441H mutations, and that protein aggregation of alpha-synuclein or tau might be secondary changes.

**Electronic supplementary material:**

The online version of this article (10.1186/s40478-018-0617-y) contains supplementary material, which is available to authorized users.

## Introduction

Parkinson’s disease (PD) is the most common neurodegenerative movement disorder. Pathologies include neuronal loss and gliosis in the substantia nigra pars compacta (SNpc), locus coeruleus (LC), and dorsal motor nucleus of the vagus nerve, as well as the appearance of Lewy pathologies [9]. Lewy pathologies are the pathological hallmark of PD, and their major component is alpha-synuclein, encoded by *synuclein alpha* (*SNCA*) [43]. Many genetic factors for PD (*PARK* from 1 to 23) have been detected in the past two decades [7]. *LRRK2* was originally mapped as a candidate region by linkage analysis from a large family living in the Sagamihara region of the Kanagawa prefecture in Japan [13]. Following its discovery, two groups concurrently reported the mutations p.R1441G, p.Y1699C, and p.R1441C in *LRRK2* from patients in England, Spain, Germany, and the United States [33, 54]. *LRRK2* is located on 12q12 (MIM#609007) and includes 51 exons, and encodes a large protein (2527 amino acids) that belongs to the ROCO protein family and includes seven domains: armadillo, ankyrin, leucine-rich repeat (LRR), Ras in complex proteins (Roc), C-terminal of Roc (COR), kinase, and WD40 [26]. Currently, seven missense mutations (p.N1437H, p.R1441C/G/H, p.Y1699C, p.G2019S, and p.I2020T) are considered pathogenic variants. *LRRK2* p.G2019S is the most common mutation, responsible for 36% of familial PD in North African Arab-Berbers, and 28% of familial PD in Ashkenazi Jews [21]. *LRRK2* p.G2385R is also known as a risk variant for the onset of PD in Asian countries such as Japan, Taiwan, and Singapore [8, 14, 46].

To date, heterogeneous brain pathology has been reported in 55 autopsies of patients harboring *LRRK2* mutations [40]. Even in the same mutation, for instance *LRRK2* p.G2019S, pathologies have been reported with and without alpha-synuclein-positive Lewy bodies or Lewy neurites, Alzheimer’s disease (AD) pathology, or tau-immunopositive glial tangle pathologies [17, 36, 38]. Neuropathology in *LRRK2* p.I2020T mutations from the original Sagamihara family also revealed heterogeneous pathologies, including pure degeneration of the SNpc without any inclusions, Lewy bodies, or multiple system atrophy (MSA) pathology [19]. It is still unclear why *LRRK2* mutations have such varied pathological and clinical manifestations.

In the present study, we detected two families (families A and B) that harbored c.4332 G > A, p.R1441H mutations in *LRRK2*, including ten PD patients presenting slowly progressing, late-onset parkinsonism. The two families had consanguinity. Our genetic analysis of seven PD patients indicated five homozygote and two heterozygote mutations of p.R1441H. Of these, we conducted brain autopsies of three patients: two homozygotes and one heterozygote. Our findings provide a new perspective of brain pathologies in patients with *LRRK2* mutations.

## Materials and methods

### Subjects

**Fig. 1:**
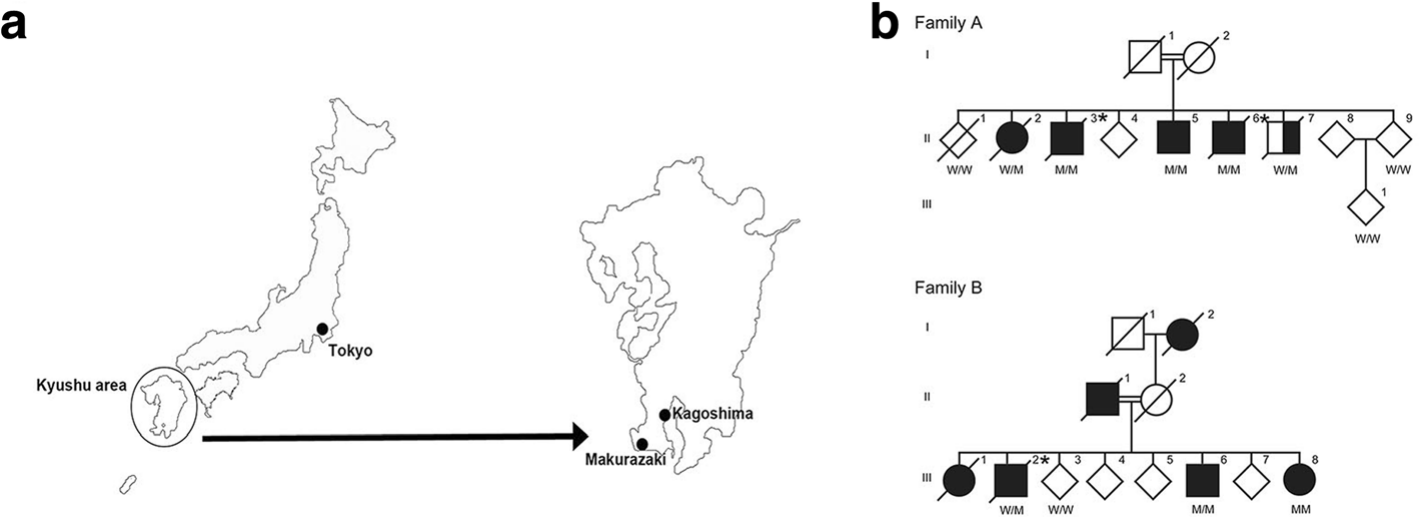
Illustrated location of Makurazaki city and the family trees of family A and B. **a** Geographical illustration of Japan. Makurazaki is a small city in the Kagoshima prefecture, located in the southernmost tip of Kyushu island. **b** Family trees of the two families harboring p.R1441H in *LRRK2* mutation and living in Makurazaki city. Parkinson’s disease is shown as black and schizophrenia is shown as half black and half white. *W*/W = wildtype, W/M = heterozygous of c.4322G > A, M/M = homozygous of c.4322G > A. Diagonal line denotes deceased individuals. Asterisk represents autopsied cases

This study was approved by the ethics committee of the Juntendo University School of Medicine, in accordance with The Code of Ethics of the World Medical Association (Declaration of Helsinki). All participants for genetic and clinical analyses gave full written informed consent before participation. The inheritance mode was defined as autosomal dominant when the family members of at least two consecutive generations were affected, and as autosomal recessive when one same-generation sibling was affected. The diagnosis of PD was established using clinical criteria [18]. A good responder to levodopa was defined as a patient whose symptoms improved with levodopa treatment. The two families, A and B, were from Makurazaki city in the Kagoshima prefecture, located in the southwest of Japan, with a population of approximately 20,000 in 2018 (Fig. 1a). The parents of each family were first cousins and born in Makurazaki. Our definition of inheritance confirmed family A as autosomal recessive inheritance, and family B as autosomal dominant inheritance (Fig. 1b).

### Genetic analyses

We collected genomic DNA using QIAamp DNA Blood Midi Kit (QIAGEN, Hilden, Germany) from eight individuals in family A, which included four patients with PD, one patient with schizophrenia without parkinsonism, and three healthy siblings. From family B we collected genomic DNA from four individuals, which included three patients with PD and one healthy sibling. We selected four patients with PD (A-II-2, A-II-6, B-III-2, and B-III-6) for whole genome sequencing (WGS). WGS was performed using TruSeq DNA PCR-Free Library Prep Kit (Illumina, San Diego, CA, USA) and paired-end sequencing (150 bp × 2) on a HiSeq X Ten (Illumina). Sequence reads from WGS were trimmed by Trimmomatic (version 0.36) [2] and aligned to the GRCh37 human reference genome using BWA (version 0.7.17) [27]. Duplicated reads were removed by Picard (https://broadinstitute.github.io/picard/). Variants calling was performed using GATK (version 4.0.1.1) [31], and variants were annotated using ANNOVAR (version 2017Jul17) [49]. *LRRK2* exon 31 was sequenced using the Sanger method, previously reported by Zimprich et al. [54]. Haplotypes were constructed using genetic markers including four SNPs and eight microsatellites mapped onto the flanking region of *LRRK2*. These genetic markers were genotyped by Sanger method or fragment analysis using fluorescence-labeled primers, as reported previously [4].

### Neuropathological analyses

We obtained brain autopsies from A-II-3, A-II-6, and B-III-2 and carried out neuropathological examinations (Fig. 1b). Brains were fixed with 15% neutral buffered formalin and the selected tissues were embedded in paraffin. The paraffin embedded blocks were sliced 6-μm thick. Brain sections were stained with hematoxylin and eosin (H&E), Klüber-Barrera (KB), methenamine-silver stain, Gallyas-Braak stain, and immunohistochemical stains using several antibodies for proteins related to neurodegenerative diseases. For immunohistochemistry, brain sections underwent antigen retrieval either by heat activation in a microwave oven or by reaction in formic acid, before being incubated overnight at 4 °C in primary antibody. The primary antibodies used were against phosphorylated alpha-synuclein (pSyn#64, monoclonal, diluted 1:10,000, Wako, Osaka, Japan), phosphorylated tau (AT8, monoclonal, diluted 1:100, Thermo Fisher Scientific, Waltham, MA, USA), amyloid beta (1–42, polyclonal, diluted 1:100, IBL, Gunma, Japan), Ubiquitin (Ubi-1, monoclonal, diluted 1:200, Abcam, Cambridge, UK), phosphorylated TAR DNA binding protein 43 (TDP43, Ser 409/410, monoclonal, diluted 1:1000, Cosmo Bio, Tokyo, Japan), LRRK2 (NB300–268, polyclonal, diluted 1:1000, Novus Biologicals, Littleton, CO, USA), tyrosine hydroxylase (TH, monoclonal, diluted 1:1000, Sigma-Aldrich, St. Louis, MO, USA), glial fibrillary acidic protein (GFAP, G-25-8-3, monoclonal, diluted 1:200, IBL), and ionized calcium-binding adapter molecule 1 (Iba1, polyclonal, diluted 1:1000, Wako) were used. Bound antibodies were visualized using the peroxidase-polymer-based method using a Histofine Simple Stain MAX-PO kit (Nichirei, Tokyo, Japan) with diaminobenzidine as the chromogen.

## Results

### Genetic analyses

We identified 13 consensus variants by WGS analysis (Table 1 and Additional file 1). Among them, 11 of the 13 variants were insertion/deletion, which might be misaligned false positive variant calls. The remaining two variants were *MUC5B* (c.7843G > A:p.G2615S) and *LRRK2* (c.4322G > A:p.R1441H). *Mucin 5B, oligomeric mucus/gelforming* (*MUC5B*) has been reported as a susceptibility gene for pulmonary fibrosis [4]. Therefore, the results of WGS indicated that *LRRK2* (c.4322G > A: p.R1441H) is a causative mutation for families A and B. Sanger sequencing validation identified eight symptomatic patients (A-II-2, A-II-3, A-II-5, A-II-6, A-II-7, B-III-2, B-III-6, and B-III-8) with p.R1441H, and four asymptomatic individuals (A-II-1, A-II-9, A-III-1, B-III-3) without p.R1441H, which signals complete segregation of p.R1441H in families A and B (Fig. 1b). In addition, Sanger sequencing revealed a homozygous mutation in five patients (A-II-3, A-II-5, A-II-6, B-III-6, and B-III-8) and a heterozygous mutation in three patients (A-II-2, A-II-7, and B-III-2; Additional file 1: Figure S1). There were no pathogenic mutations, as well as risk variants and haplotypes, including *SNCA*, *PAKR16*, *BST1*, and *MAPT*, related to familial PD except *LRRK2* p.R1441H in our WGS reads. Haplotype analysis indicated that patients from families A and B shared a common haplotype in the region of between D12S2080 and D12S2522, which signals a founder effect (Additional file 1: Table S2).

**Table 1:** Variant filtering of whole genome sequencing (WGS) reads

	Family A		Family B	
ID	A-II-2	A-II-6	B-III-2	B-III-6
Phenotype	PD	PD	PD	PD
Mean depth of coverage	31.30	31.66	29.65	33.11
Exonic or splicing variants	20,330	20,417	20,594	20,680
Frequency < 0.0001	245	260	286	244
Consensus variants of each families	89		85	
Consensus variants of all subjects	13			

The variants detected in WGS were filtered using our criteria: (1) located in exons or splicing sites; (2) frequencies from variant databases (ExAC, Exome Variant Server, and HGVD) less than 0.0001. Consensus variants were selected regardless of zygosity

### Case presentations

The parents of family A (A-I-1 and A-I-2) and family B (B-II-1 and B-II-2) married with consanguinity. All patients indicated as black symbols in the family trees were clinically diagnosed with typical PD (Fig. 1b). A-II-7 was diagnosed as schizophrenia without parkinsonism. Age at onset of PD was 61.25 ± 9.19 (± SD) in all patients, 61.60 ± 7.23 in patients with homozygous mutations, and 68.50 ± 6.54 in patients with heterozygous mutations. There were no differences in age at onset between homozygous and heterozygous mutations (*p* > 0.05). The clinical history of the three autopsied cases is described in the following sections.

### A-II-3

This patient had a homozygous mutation of p.R1441H in *LRRK2*. At 68 years of age, he noticed left-side dominant tremor in his upper and lower limbs. The attending doctor observed his rigidity, tremor, and masked face and diagnosed him with PD. Following diagnosis, his motor symptoms showed a good response to levodopa carbidopa 200 mg and cabergoline 2 mg per day. In the following few years, he remained in good condition and with good activity of daily living, without support from anyone. At 73 years of age, the wearing off phenomenon emerged and at 74 years of age, he died suddenly at home.

### A-II-6

This patient had a homozygous mutation of p.R1441H in *LRRK2*. At the age of 60 years, he noticed resting tremor in his right upper and lower limbs. The next year, he went to the local hospital and was diagnosed with PD, and had a good response to levodopa/benserazide, trihexyphenidyl, and pramipexole. His parkinsonism slowly progressed, and by 72 years of age, visual and auditory hallucinations, diurnal fluctuations, dyskinesia, and camptocormia became prominent. At 74 years of age, he was bed-ridden. At his final admission at Juntendo Hospital, his intelligence was well preserved, with a Mini-Mental State Examination (MMSE) score of 26/30. He exhibited marked fluctuations in consciousness, bradyphrenia, visual hallucinations, hypersexuality, and delusions of persecution. We observed disuse-induced atrophy in the upper and lower limbs, and contracture and pes equinus in the lower limbs, due to the long duration of his bed-ridden state. Deep tendon reflexes were decreased in the upper and lower limbs, and dysautonomia such as orthostatic hypotension, urinary incontinence, and constipation was not seen. Rigidity and tremor were mild even at the end stage. Brain magnetic resonance imaging (MRI) indicated no abnormalities. ^123^I-metaiodobenzilguanidine (MIBG) myocardial scintigraphy revealed no decreasing rate with 3.00 at early and 3.40 at delay of heart to mediastinum (H/M) ratio (normal value: over 2.2). Dopamine transporter (DAT) imaging showed a severe decrease in DAT densities. A nerve conduction study indicated axonal polyneuropathy. At the age of 74 years, the patient died from aspiration pneumonia.

### B-iii-2

This patient had a heterozygous mutation of p.R1441H in *LRRK2*. At the age of 72, he became aware of a resting tremor in his right extremities. He was diagnosed with PD, and presented a good response to levodopa/benserazide, entacapone, and selegiline over 10 years. At the age of 85, wearing off was prominent, but he could walk alone. At 87 years of age, he found walking difficult after the onset of a lumbar fracture. Following this injury, he repeatedly experienced aspiration pneumonia. In the same year, his consciousness was disturbed due to hypoxia and aspiration pneumonia, and he was admitted to the hospital. After comprehensive treatment, he died from the progression of pneumonia, multiple organ failure, and hypoxia. At his final hospital admission, delusion was present, but cognitive function was preserved (MMSE: 25/30). His brain MRI indicated no abnormalities, while his ^123^I-MIBG myocardial scintigraphy indicated normal uptake, with 2.51 in early and 2.79 in delay of H/M ratio. A DAT scan demonstrated a severe decrease in uptake with left-side-dominant laterality. A nerve conduction study indicated axonal polyneuropathy.

### Pathological analysis

The neuropathological findings of three patients (individual A-II-3, A-II-6, and B-III-2) are presented. The brain weights of A-II-3, A-II-6, and B-III-2 were 1350 g, 1350 g, and 1126 g, respectively.

**Fig. 2:**
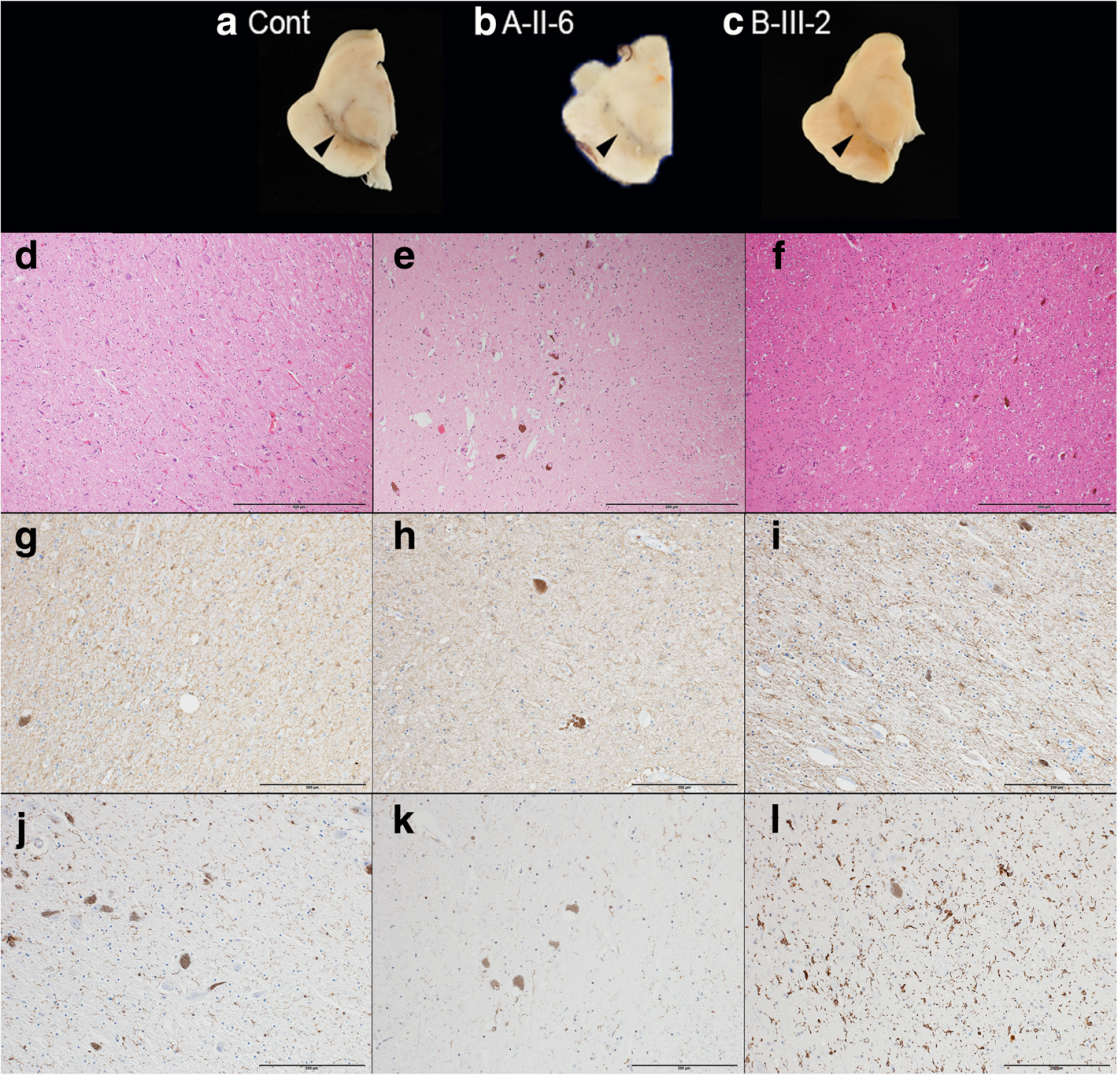
Neuronal loss and astrogliosis in the substantia nigra of cases with the *LRRK2* p.R1441H mutation. A macroscopic picture of the midbrains (**a**: age-matched control, **b:** A-II-6, **c**: B-III-2). The substantia nigra of A-II-6 and B-III-2 were markedly depigmented, compared with the age-matched control (a macroscopic photograph of A-II-3 was not available). Black arrowheads indicate the pigmented nigral areas. The substantia nigra of all cases showed apparent neuronal loss and astrogliosis in hematoxylin and eosin (H&E) stain (**d**: A-II-3, **e**: A-II-6, **f**: B-III-2) and glial fibrillary acidic protein (GFAP) stain (**g**: A-II-3, **h**: A-II-6, **i**: B-III-2), but proliferations of reactive microglia were absent or mild (**j**: A-II-3, **k**: A-II-6, **l**: B-III-2). The scale bars represent d–f: 500 μm, g–i: 200 μm, and j–l: 200 μm, respectively

**Fig. 3:**
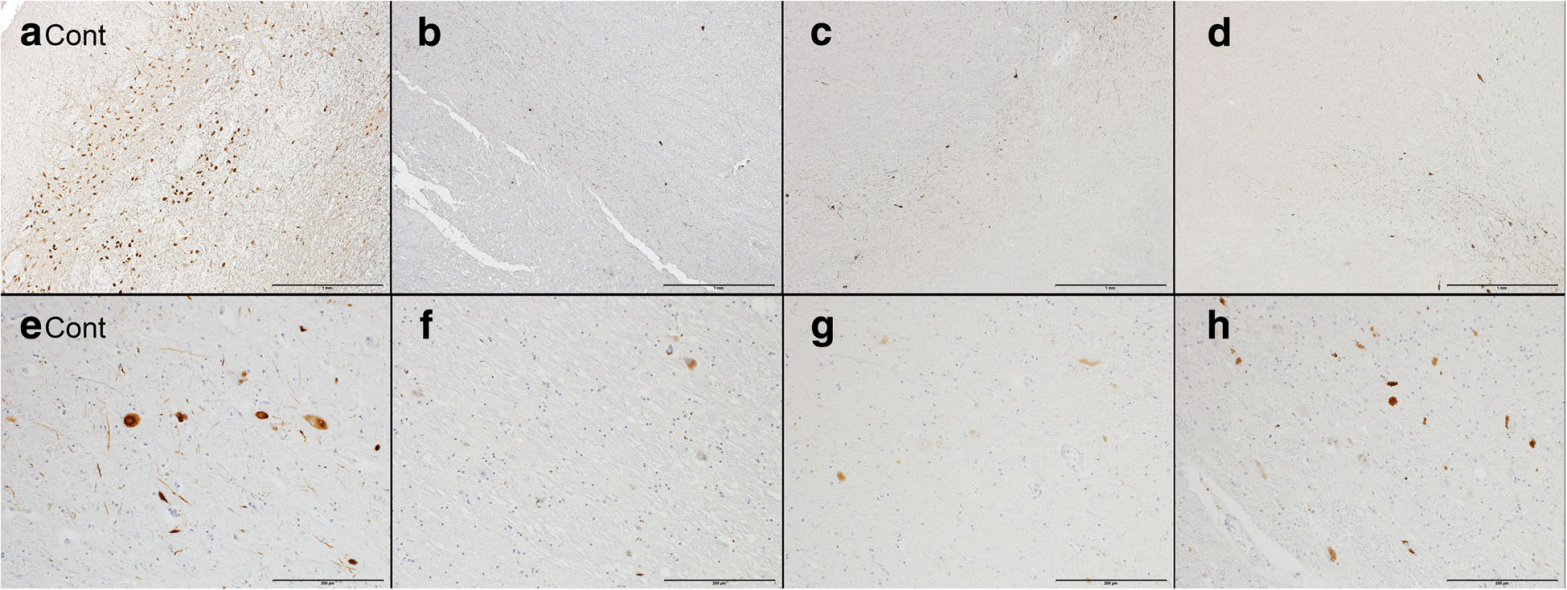
Dopaminergic cell loss and absence of alpha-synuclein pathologies in the substantia nigra of cases with the *LRRK2* p.R1441H mutation. Severe losses of dopaminergic neurons were present in the substantia nigra of cases with the mutation, compared with age-matched controls, using a tyrosine hydroxylase (TH) immunostain (**a**: age-matched control, **b**: A-II-3, **c**: A-II-6, **d**: B-III-2). However, sporadic PD with Lewy pathology corresponding to Braak stage 5 showed typical alpha-synuclein-positive neuronal inclusions in the substantia nigra; there was no Lewy pathology in the substantia nigra or other brain regions of cases with the mutation, as assessed with immunohistochemical analysis for alpha-synuclein (**e**: age-matched control, **f**: A-II-3, **g**: A-II-6, **h**: B-III-2). The scale bars represent a–d: 1 mm, and e–h: 200 μm, respectively

Macroscopically, the substantia nigra (SN) was markedly depigmented in our cases, compared with age-matched controls (Fig. 2 a-c), but the LC was preserved. Atrophy and morphological changes were not observed in other brain regions. In H&E, KB, GFAP, and IBA1 staining, neuronal cell loss and astrogliosis were apparent in the SN (Fig. 2 d–i). Almost all microglia exhibited a ramified shape (resting form) in the SN of all three cases; small numbers of ameboid-shaped cells (reactive form) appeared only in B-III-2 (Fig. 2 j–l). These findings were considered common pathologies of chronic nigral degeneration. In TH stains, loss of dopaminergic neurons was apparent in the SNpc, compared with age matched-controls (Fig. 3 a–d). In contrast, there was no neuronal cell loss or gliosis in the LC in any case. The remains of neurons showed no Lewy bodies in H&E sections. In anti-phosphorylated alpha-synuclein (p-alpha-synuclein) and ubiquitin immunostains, there were no Lewy pathologies (bodies, neurites, or dots) in the SN, compared with sporadic PD (Fig. 3 e–h). Moreover, p-alpha-synuclein-positive pathologies were not detected at all in the brainstem, limbic area, subcortical nuclei, white matter, or neocortex in any individual. Phosphorylated-tau (p-tau)-positive neurofibrillary tangles (NFT) and threads were limited to within the parahippocampus and hippocampus (Braak NFT stage II) [3]. Amyloid-beta-positive senile plaques were detected within the range of normal aging in the neocortex. These tau pathologies were considered primary age-related tauopathy (PART) [6]. There were no TDP43-positive pathologies or LRRK2-positive pathologies. These pathological findings were common to all three patients. In addition to these common findings, a small number of p-tau-positive structures were observed in the brainstem and cerebellar dentate nucleus of individual B-III-2, while the peripheral nerve (sural nerve) was also evaluated in A-II-6 and demonstrated preserved axons and myelin. In summary, the major pathological feature of all three individuals was isolated nigral degeneration without Lewy or other pathological inclusions.

## Discussion

In the present study, we investigated two families that harbored homozygous and heterozygous mutations of p.R1441H in *LRRK2.* All affected members had a founder effect, suggesting a common haplotype in two different families. The two families came from a narrow district in Makurazaki city, with consanguinity. The environment incidentally generated both heterozygous and homozygous mutations of p.R1441H in two families. All PD patients presented late-onset parkinsonism with a good response to levodopa and a mild disease course, without apparent cognitive decline or dysautonomia. The clinical manifestations were similar to those in patients with sporadic PD or PD with other *LRRK2* mutations. Most PD patients with *LRRK2* mutations are heterozygous missense mutations with an autosomal dominant inheritance mode, which we infer as a gain-of-toxic-function pathological mechanism of *LRRK2* mutations. In the families in our study, there were no differences in clinical symptoms, age at onset, or brain pathologies between patients with heterozygous and homozygous mutations. Previous reports also described no apparent differences in symptoms between homozygous and heterozygous mutations in *LRRK2* p.G2019S [1, 24, 25]. The patients with *LRRK2* p.G2019S homozygous mutations had middle-aged onset, tremor-dominant and mixed type of tremor, and rigid akinesia, similar to heterozygous patients [24]. It has also been reported that homozygous p.R1441C knock-in mice present a normal phenotype, with no dopaminergic neurodegeneration [48]. It remains unclear why homozygote and heterozygote patients with *LRRK2* mutations have similar clinical manifestations.

Arginine (R) is located at position 1441within the Roc domain of the LRRK2 protein. The missense mutation in R1441 induces different amino acid changes of glycine (G), cysteine (C), or histidine (H) [30, 33, 54]. *LRRK2* mutations of p.R1441C/G/H induce late-onset parkinsonism with good response to levodopa, and closely resemble sporadic PD or PD with *LRRK2* p.G2019S mutations [20, 30, 34]. The p.R1441H mutations have been identified in four families from Asia, Europe, and North America; it is present in diverse ethnicities [12, 30, 42, 53]. One rare case with *LRRK2* p.R1441H has been related to progressive supranuclear palsy [42]. However, our three autopsy cases showed homogeneity in their symptoms; they had preserved cognitive function until the end of their lives, with the appearance of persistent psychosis in the advanced stage, and without dysautonomia. These findings may arise from their consanguinity, a founder effect, and their descent from a small city population.

A summary of 55 autopsied cases with *LRRK2* mutations (p.G2019S, p.I2020T, p.R1441C, and others) has been previously reported [40]. Most pathologies involved SNpc degeneration associated with Lewy pathologies. However, other patients presented broad types of pathology, including pure nigral degeneration without any inclusions, MSA-like-synucleinopathy, PSP-like tauopathy, frontotemporal lobar degeneration with ubiquitin-positive-inclusions, and coexistence with TDP-43-positive inclusions. The pathological heterogeneity suggests that *LRRK2* mutations may cause a cascade of effects to induce neuronal deterioration.

To our knowledge, this study is the first report of neuropathology in *LRRK2* p.R1441H mutations. In addition, there have been no pathological reports of any homozygous mutations in *LRRK2*. Our pathological presentation of both homozygous and heterozygous mutations in *LRRK2* is crucial for considering LRRK2-associated pathogenesis. Isolated nigral (SNpc) degeneration without alpha-synuclein, tau, amyloid-beta, ubiquitin, and TDP43 pathology was a prominent feature in all three of our autopsy cases. Previous studies have reported pure nigral degeneration, without inclusions of disease-specific proteins, in p.G2019S, p.R1441G, and p.I2020T mutations [15, 17, 19, 29]. In addition, all patients showed typical sporadic PD-like parkinsonism, similar to our cases. Considering the clinicopathological relationship, their parkinsonism might be caused by neurodegeneration of the SNpc, irrespective of expression levels of alpha-synuclein pathologies. Nigral degeneration not associated with Lewy pathologies was also observed in the brains of most *parkin RBR E3 ubiquitin protein ligase (PRKN*) mutations and one *PTEN induced putative kinase 1* (*PINK1*) mutation previously [10, 45]. Parkin and PINK1 collaborate in mitophagy to remove damaged mitochondria and maintain mitochondrial quality [44]. There is convincing evidence linking LRRK2 with autophagy/mitophagy [5, 23, 32, 37, 39, 47], with regulating roles for LRRK2 involving mTOR signaling [11, 22, 28, 39, 52] as well as Parkin/PINK1 mitophagy. In contrast, LRRK2, a large multi-domain protein, plays an important role in phosphorylation of itself and of target substrates in cellular signaling. Some mutations in the kinase domain, especially p.G2019S, are considered to augment kinase activity, and excess kinase activity is known to induce neurotoxicity [41, 50]. Alternatively, mutations in the Roc-COR domain can cause a decrease in GTPase activity and are thought to play an important role in neurodegeneration [51]. In addition, in relation to alpha-synuclein and tau pathologies, several studies have shown that LRRK2 affects these protein aggregations, but alpha-synuclein- or tau-aggregation-related pathogenesis in brains with *LRRK2* mutations remains poorly understood [26]. Our three cases, and twelve previously reported cases (p.R1441H and p.R1441G in the Roc domain, p.G2019S and p.I2020T in the kinase domain), all showed SNpc degeneration without Lewy or other pathological inclusions, although these enzymes act at a steady rate and the biological aberrations induced by these mutations are quite different. Recently, a linkage between Ras analogue in brain (Rab) GTPases and alpha-synuclein, LRRK2, and vacuolar sorting protein (VPS) 35 associations with PD pathogenesis was highlighted [16]. One study reported that p.R1441C in the GTPase domain enhances GTP binding and stimulates LRRK2 activity through interaction with Rab29 and the Golgi apparatus [35]. These data suggest that p.R1441H in the GTPase domain can also cause LRRK2-associated neurotoxicity induced by Rab29-mediated Golgi recruitment.

## Conclusions

It was noteworthy that our three autopsied series showed homogeneous pathology. Regardless of the homozygous or heterozygous mutations of p.R1441H, the pathological findings and the clinical features were similar, suggesting that any possibility of a gene dosage effect could be excluded. Two consanguineous families with *LRRK2* p.R1441H had a founder effect; the similarity might be caused by their homogeneous genetic background. Our pathological findings indicated that isolated nigral degeneration is essential pathology for *LRRK2* p.R1441H mutations. When considering previous reports of neuropathologies from *LRRK2* mutations, SNpc degeneration was a constant finding in all cases. Other findings of protein pathologies, such as alpha-synuclein, tau, ubiquitin, and TDP43, were inconsistent. Thus, we consider that neuronal degeneration in the SNpc is primary degeneration, caused by neurotoxicity of pathogenic *LRRK2* mutants. We speculate that abnormal protein aggregation, such as of alpha-synuclein, tau, ubiquitin, or TDP43, would be downstream in the pathological cascade of LRRK2. Our three autopsy cases strongly support this theory, showing isolated nigral degeneration with *LRRK2* mutation. In addition, it is important to investigate the possibility of proteopathic aggregations that have not been identified previously in *LRRK2* mutated pathologies, in future pathological and biochemical studies.

## Additional file

Additional file 1: **Table S1**. Consensus nonsynonymous variants detected by whole genome sequencing. A portion of the output from ANNOVAR is shown. Most of the variants are indel and might be misaligned variant calls. NA: not applicable. **Figure S1**. Sequence electropherogram of *LRRK2* c.4322G > A:p.R1441H. Three representative sequences are shown. Healthy sibling A-II-1 has wild type (*W*/W), patient B-III-2 has heterozygous (W/M), and patient A-II-3 has homozygous (M/M) c.4322G > A variant (arrowhead) in *LRRK2* exon 31. **Table S2** Comparison of haplotypes of the *LRRK2* region. All patients of families A and B harboring *LRRK2* c.4322G > A:p.R1441H shared a common haplotype from D12S2080 to D12S2522 (in bold). (DOCX 365 kb)

## Abbreviations

AD: Alzheimer’s disease
COR: C-terminal of Roc
DAT: Dopamine transporter
GFAP: Glial fibrillary acidic protein
H&E: Hematoxylin and eosin
H/M: Heart to mediastinum
Iba1: Ionized calcium-binding adapter molecule 1
KB: Klüber-Barrera
LC: Locus coeruleus
LRR: leucine-rich repeat
LRRK2: Leucine-rich repeat kinase 2
MIBG: Metaiodobenzilguanidine
MMSE: Mini-Mental State Examination
MRI: Magnetic resonance imaging
MSA: Multiple system atrophy
MUC5B: Mucin 5B, oligomeric mucus/gelforming
NFT: neurofibrillary tangles
P-alpha-synuclein: Phosphorylated alpha-synuclein
PART: primary age-related tauopathy
PD: Parkinson’s disease
PINK1: PTEN induced putative kinase 1
PRKN: parkin RBR E3 ubiquitin protein ligase
P-tau: Phosphorylated-tau
Rab: Ras analogue in brain
Roc: Ras in complex proteins
SN: Substantia nigra
SNCA: synuclein alpha
SNpc: Substantia nigra pars compacta
TDP43: TAR DNA binding protein 43
TH: Tyrosine hydroxylase
VPS: Vacuolar sorting protein
WGS: Whole genome sequencing

## References

[1] Alcalay Roy N., Mirelman Anat, Saunders-Pullman Rachel, Tang Ming-X, Mejia Santana Helen, Raymond Deborah, Roos Ernest, Orbe-Reilly Martha, Gurevich Tanya, Bar Shira Anat, Gana Weisz Mali, Yasinovsky Kira, Zalis Maayan, Thaler Avner, Deik Andres, Barrett Matthew James, Cabassa Jose, Groves Mark, Hunt Ann L., Lubarr Naomi, San Luciano Marta, Miravite Joan, Palmese Christina, Sachdev Rivka, Sarva Harini, Severt Lawrence, Shanker Vicki, Swan Matthew Carrington, Soto-Valencia Jeannie, Johannes Brooke, Ortega Robert, Fahn Stanley, Cote Lucien, Waters Cheryl, Mazzoni Pietro, Ford Blair, Louis Elan, Levy Oren, Rosado Llency, Ruiz Diana, Dorovski Tsvyatko, Pauciulo Michael, Nichols William, Orr-Urtreger Avi, Ozelius Laurie, Clark Lorraine, Giladi Nir, Bressman Susan, Marder Karen S. (2013). Parkinson disease phenotype in Ashkenazi jews with and withoutLRRK2G2019S mutations. Movement Disorders.

[2] Bolger AM, Lohse M, Usadel B (2014). Trimmomatic: a flexible trimmer for Illumina sequence data. Bioinformatics.

[3] Braak H, Braak E (1991). Neuropathological stageing of Alzheimer-related changes. Acta Neuropathol.

[4] Chandra S, Chen X, Rizo J, Jahn R, Sudhof TC (2003). A broken alpha -helix in folded alpha -Synuclein. J Biol Chem.

[5] Cherra SJ, Steer E, Gusdon AM, Kiselyov K, Chu CT (2013). Mutant LRRK2 elicits calcium imbalance and depletion of dendritic mitochondria in neurons. Am J Pathol.

[6] Crary JF, Trojanowski JQ, Schneider JA, Abisambra JF, Abner EL, Alafuzoff I, Arnold SE, Attems J, Beach TG, Bigio EH (2014). Primary age-related tauopathy (PART): a common pathology associated with human aging. Acta Neuropathol.

[7] Deng H, Wang P, Jankovic J (2018). The genetics of Parkinson disease. Ageing Res Rev.

[8] Di Fonzo A, Wu-Chou YH, Lu CS, van Doeselaar M, Simons EJ, Rohe CF, Chang HC, Chen RS, Weng YH, Vanacore N (2006). A common missense variant in the LRRK2 gene, Gly2385Arg, associated with Parkinson's disease risk in Taiwan. Neurogenetics.

[9] Dickson DW (2018). Neuropathology of Parkinson disease. Parkinsonism Relat Disord.

[10] Doherty KM, Hardy J (2013). Parkin disease and the Lewy body conundrum. Mov Disord.

[11] Ferree A, Shirihai O (2012). Mitochondrial dynamics: the intersection of form and function. Adv Exp Med Biol.

[12] Ferreira JJ, Guedes LC, Rosa MM, Coelho M, van Doeselaar M, Schweiger D, Di Fonzo A, Oostra BA, Sampaio C, Bonifati V (2007). High prevalence of LRRK2 mutations in familial and sporadic Parkinson’s disease in Portugal. Mov Disord.

[13] Funayama M, Hasegawa K, Kowa H, Saito M, Tsuji S, Obata F (2002). A new locus for Parkinson's disease (PARK8) maps to chromosome 12p11.2-q13.1. Ann Neurol.

[14] Funayama M, Li Y, Tomiyama H, Yoshino H, Imamichi Y, Yamamoto M, Murata M, Toda T, Mizuno Y, Hattori N (2007). Leucine-rich repeat kinase 2 G2385R variant is a risk factor for Parkinson disease in Asian population. Neuroreport.

[15] Gaig C, Marti MJ, Ezquerra M, Rey MJ, Cardozo A, Tolosa E (2007). G2019S LRRK2 mutation causing Parkinson's disease without Lewy bodies. J Neurol Neurosurg Psychiatry.

[16] Gao Y, Wilson GR, Stephenson SEM, Bozaoglu K, Farrer MJ, Lockhart PJ (2018). The emerging role of Rab GTPases in the pathogenesis of Parkinson's disease. Mov Disord.

[17] Giasson BI, Covy JP, Bonini NM, Hurtig HI, Farrer MJ, Trojanowski JQ, Van Deerlin VM (2006). Biochemical and pathological characterization of Lrrk2. Ann Neurol.

[18] Gibb WR, Lees AJ (1988). The relevance of the Lewy body to the pathogenesis of idiopathic Parkinson's disease. J Neurol Neurosurg Psychiatry.

[19] Hasegawa K, Stoessl AJ, Yokoyama T, Kowa H, Wszolek ZK, Yagishita S (2009). Familial parkinsonism: study of original Sagamihara PARK8 (I2020T) kindred with variable clinicopathologic outcomes. Parkinsonism Relat Disord.

[20] Haugarvoll K, Rademakers R, Kachergus JM, Nuytemans K, Ross OA, Gibson JM, Tan EK, Gaig C, Tolosa E, Goldwurm S (2008). Lrrk2 R1441C parkinsonism is clinically similar to sporadic Parkinson disease. Neurology.

[21] Healy DG, Falchi M, O'Sullivan SS, Bonifati V, Durr A, Bressman S, Brice A, Aasly J, Zabetian CP, Goldwurm S (2008). Phenotype, genotype, and worldwide genetic penetrance of LRRK2-associated Parkinson’s disease: a case-control study. Lancet Neurol.

[22] Herzig MC, Kolly C, Persohn E, Theil D, Schweizer T, Hafner T, Stemmelen C, Troxler TJ, Schmid P, Danner S (2011). LRRK2 protein levels are determined by kinase function and are crucial for kidney and lung homeostasis in mice. Hum Mol Genet.

[23] Hindle SJ, Elliott CJ (2013). Spread of neuronal degeneration in a dopaminergic, Lrrk-G2019S model of Parkinson disease. Autophagy.

[24] Hulihan MM, Ishihara-Paul L, Kachergus J, Warren L, Amouri R, Elango R, Prinjha RK, Upmanyu R, Kefi M, Zouari M (2008). LRRK2 Gly2019Ser penetrance in Arab-Berber patients from Tunisia: a case-control genetic study. Lancet Neurol.

[25] Ishihara L, Warren L, Gibson R, Amouri R, Lesage S, Durr A, Tazir M, Wszolek ZK, Uitti RJ, Nichols WC (2006). Clinical features of Parkinson disease patients with homozygous leucine-rich repeat kinase 2 G2019S mutations. Arch Neurol.

[26] Islam MS, Moore DJ (2017). Mechanisms of LRRK2-dependent neurodegeneration: role of enzymatic activity and protein aggregation. Biochem Soc Trans.

[27] Li H, Durbin R (2009). Fast and accurate short read alignment with burrows-wheeler transform. Bioinformatics.

[28] Manzoni C, Mamais A, Dihanich S, Abeti R, Soutar MPM, Plun-Favreau H, Giunti P, Tooze SA, Bandopadhyay R, Lewis PA (2013). Inhibition of LRRK2 kinase activity stimulates macroautophagy. Biochim Biophys Acta.

[29] Marti-Masso JF, Ruiz-Martinez J, Bolano MJ, Ruiz I, Gorostidi A, Moreno F, Ferrer I, Lopez de Munain A (2009). Neuropathology of Parkinson’s disease with the R1441G mutation in LRRK2. Mov Disord.

[30] Mata IF, Kachergus JM, Taylor JP, Lincoln S, Aasly J, Lynch T, Hulihan MM, Cobb SA, Wu RM, Lu CS (2005). Lrrk2 pathogenic substitutions in Parkinson’s disease. Neurogenetics.

[31] McKenna A, Hanna M, Banks E, Sivachenko A, Cibulskis K, Kernytsky A, Garimella K, Altshuler D, Gabriel S, Daly M (2010). The Genome Analysis Toolkit: a MapReduce framework for analyzing next-generation DNA sequencing data. Genome Res.

[32] Orenstein SJ, Kuo SH, Tasset I, Arias E, Koga H, Fernandez-Carasa I, Cortes E, Honig LS, Dauer W, Consiglio A (2013). Interplay of LRRK2 with chaperone-mediated autophagy. Nat Neurosci.

[33] Paisan-Ruiz C, Jain S, Evans EW, Gilks WP, Simon J, van der Brug M, Lopez de Munain A, Aparicio S, Gil AM, Khan N (2004). Cloning of the gene containing mutations that cause PARK8-linked Parkinson’s disease. Neuron.

[34] Paisan-Ruiz C, Lewis PA, Singleton AB (2013). LRRK2: cause, risk, and mechanism. J Parkinsons Dis.

[35] Purlyte E, Dhekne HS, Sarhan AR, Gomez R, Lis P, Wightman M, Martinez TN, Tonelli F, Pfeffer SR, Alessi DR (2018). Rab29 activation of the Parkinson’s disease-associated LRRK2 kinase. EMBO J.

[36] Rajput A, Dickson DW, Robinson CA, Ross OA, Dachsel JC, Lincoln SJ, Cobb SA, Rajput ML, Farrer MJ (2006). Parkinsonism, Lrrk2 G2019S, and tau neuropathology. Neurology.

[37] Ramonet D, Daher JP, Lin BM, Stafa K, Kim J, Banerjee R, Westerlund M, Pletnikova O, Glauser L, Yang L (2011). Dopaminergic neuronal loss, reduced neurite complexity and autophagic abnormalities in transgenic mice expressing G2019S mutant LRRK2. PLoS One.

[38] Ross OA, Toft M, Whittle AJ, Johnson JL, Papapetropoulos S, Mash DC, Litvan I, Gordon MF, Wszolek ZK, Farrer MJ (2006). Lrrk2 and Lewy body disease. Ann Neurol.

[39] Schapansky J, Nardozzi JD, Felizia F, LaVoie MJ (2014). Membrane recruitment of endogenous LRRK2 precedes its potent regulation of autophagy. Hum Mol Genet.

[40] Schneider SA, Alcalay RN (2017). Neuropathology of genetic synucleinopathies with parkinsonism: review of the literature. Mov Disord.

[41] Smith WW, Pei Z, Jiang H, Dawson VL, Dawson TM, Ross CA (2006). Kinase activity of mutant LRRK2 mediates neuronal toxicity. Nat Neurosci.

[42] Spanaki C, Latsoudis H, Plaitakis A (2006). LRRK2 mutations on Crete: R1441H associated with PD evolving to PSP. Neurology.

[43] Spillantini MG, Schmidt ML, Lee VM, Trojanowski JQ, Jakes R, Goedert M (1997). Alpha-synuclein in Lewy bodies. Nature.

[44] Stolz A, Dikic I (2014). PINK1-PARKIN interplay: down to ubiquitin phosphorylation. Mol Cell.

[45] Takanashi M, Li Y, Hattori N (2016). Absence of Lewy pathology associated with PINK1 homozygous mutation. Neurology.

[46] Tan EK, Zhao Y, Skipper L, Tan MG, Di Fonzo A, Sun L, Fook-Chong S, Tang S, Chua E, Yuen Y (2007). The LRRK2 Gly2385Arg variant is associated with Parkinson's disease: genetic and functional evidence. Hum Genet.

[47] Tong Y, Giaime E, Yamaguchi H, Ichimura T, Liu Y, Si H, Cai H, Bonventre JV, Shen J (2012). Loss of leucine-rich repeat kinase 2 causes age-dependent bi-phasic alterations of the autophagy pathway. Mol Neurodegener.

[48] Tong Y, Pisani A, Martella G, Karouani M, Yamaguchi H, Pothos EN, Shen J (2009). R1441C mutation in LRRK2 impairs dopaminergic neurotransmission in mice. Proc Natl Acad Sci U S A.

[49] Wang K, Li M, Hakonarson H (2010). ANNOVAR: functional annotation of genetic variants from high-throughput sequencing data. Nucleic Acids Res.

[50] West AB, Moore DJ, Biskup S, Bugayenko A, Smith WW, Ross CA, Dawson VL, Dawson TM (2005). Parkinson’s disease-associated mutations in leucine-rich repeat kinase 2 augment kinase activity. Proc Natl Acad Sci U S A.

[51] Xiong Y, Coombes CE, Kilaru A, Li X, Gitler AD, Bowers WJ, Dawson VL, Dawson TM, Moore DJ (2010). GTPase activity plays a key role in the pathobiology of LRRK2. PLoS Genet.

[52] Yakhine-Diop SM, Bravo-San Pedro JM, Gomez-Sanchez R, Pizarro-Estrella E, Rodriguez-Arribas M, Climent V, Aiastui A, Lopez de Munain A, Fuentes JM, Gonzalez-Polo RA (2014). G2019S LRRK2 mutant fibroblasts from Parkinson’s disease patients show increased sensitivity to neurotoxin 1-methyl-4-phenylpyridinium dependent of autophagy. Toxicology.

[53] Zabetian CP, Samii A, Mosley AD, Roberts JW, Leis BC, Yearout D, Raskind WH, Griffith A (2005). A clinic-based study of the LRRK2 gene in Parkinson disease yields new mutations. Neurology.

[54] Zimprich A, Biskup S, Leitner P, Lichtner P, Farrer M, Lincoln S, Kachergus J, Hulihan M, Uitti RJ, Calne DB (2004). Mutations in LRRK2 cause autosomal-dominant parkinsonism with pleomorphic pathology. Neuron.

