# Supplementary material for: Isolated nigral degeneration without pathological protein aggregation in autopsied brains with LRRK2 p.R1441H homozygous and heterozygous mutations
Source: Acta Neuropathol Commun. 2018 Oct 17;6:105. doi: 10.1186/s40478-018-0617-y (PMC6192197; doi:10.1186/s40478-018-0617-y)

**Supplementary data**

**Supplementary Table 1; Consensus nonsynonymous variants detected by whole genome sequencing.**

Part of output from ANNOVAR was shown. most of the variants indel and it might be misaligned variant calls. NA: not applicable.

| Chromosome | Start | End | Gene | Reference | Alteration | snp138 | | Amino acid Change |  |
| --- | --- | --- | --- | --- | --- | --- | --- | --- | --- |
| 2 | 37468887 | 37468887 | NDUFAF7 | C | - | NA | | NDUFAF7:NM_144736:exon5:c.575delC:p.S192fs |  |
| 2 | 37468889 | 37468892 | NDUFAF7 | GGGA | - | | NA | NDUFAF7:NM_144736:exon5:c.577_580del:p.G193fs | |
| 2 | 37468895 | 37468898 | NDUFAF7 | CCAA | - | | NA | NDUFAF7:NM_144736:exon5:c.583_586del:p.P195fs | |
| 2 | 37468907 | 37468907 | NDUFAF7 | - | ACGGCGACC | | NA | NDUFAF7:NM_144736:exon5:c.595_596insACGGCGACC:p.Y199delinsYGDH | |
| 3 | 42251577 | 42251577 | TRAK1 | - | GGA | | rs10634555 | TRAK1:NM_001265609:exon13:c.1841_1842insGGA:p.T614delinsTE,TRAK1:NM_014965:exon13:c.1889_1890insGGA:p.T630delinsTE,TRAK1:NM_001265608:exon14:c.2063_2064insGGA:p.T688delinsTE | |
| 3 | 42251577 | 42251577 | TRAK1 | - | GGAGGA | | NA | TRAK1:NM_001265609:exon13:c.1841_1842insGGAGGA:p.T614delinsTEE,TRAK1:NM_014965:exon13:c.1889_1890insGGAGGA:p.T630delinsTEE,TRAK1:NM_001265608:exon14:c.2063_2064insGGAGGA:p.T688delinsTEE | |
| 6 | 170627695 | 170627695 | FAM120B | - | CCCTGAACCCAGGCAAGAAGTTCCCATGTGTACAGG | | NA | FAM120B:NM_001286379:exon2:c.1253_1254insCCCTGAACCCAGGCAAGAAGTTCCCATGTGTACAGG:p.D418delinsDPEPRQEVPMCTG,FAM120B:NM_001286380:exon2:c.1286_1287insCCCTGAACCCAGGCAAGAAGTTCCCATGTGTACAGG:p.D429delinsDPEPRQEVPMCTG,FAM120B:NM_032448:exon2:c.1217_1218insCCCTGAACCCAGGCAAGAAGTTCCCATGTGTACAGG:p.D406delinsDPEPRQEVPMCTG | |
| 7 | 150783920 | 150783922 | AGAP3 | TCT | - | | NA | AGAP3:NM_001042535:exon1:c.92_94del:p.31_32del,AGAP3:NM_001308304:exon1:c.92_94del:p.31_32del,AGAP3:NM_031946:exon1:c.92_94del:p.31_32del | |
| 7 | 150783922 | 150783922 | AGAP3 | - | GGGG | | NA | AGAP3:NM_001042535:exon1:c.94_95insGGGG:p.C32fs,AGAP3:NM_001308304:exon1:c.94_95insGGGG:p.C32fs,AGAP3:NM_031946:exon1:c.94_95insGGGG:p.C32fs | |
| 11 | 1265953 | 1265953 | MUC5B | G | A | | rs183370753 | MUC5B:NM_002458:exon31:c.7843G>A:p.G2615S | |
| 12 | 25261759 | 25261759 | CASC1 | - | AAAAAAAAAAAAAAAAAAAAAA | | NA | NA | |
| 12 | 40704237 | 40704237 | LRRK2 | G | A | | rs34995376 | LRRK2:NM_198578:exon31:c.4322G>A:p.R1441H | |
| 14 | 92537354 | 92537354 | ATXN3 | - | TGCTGCTGCTGCTGCTGCTGCTGCTGCTGCTGCTGCTGCTGCTGCTGCTG | | NA | ATXN3:NM_001164782:exon2:c.67_68insCAGCAGCAGCAGCAGCAGCAGCAGCAGCAGCAGCAGCAGCAGCAGCAGCA:p.G23fs,ATXN3:NM_001164774:exon3:c.232_233insCAGCAGCAGCAGCAGCAGCAGCAGCAGCAGCAGCAGCAGCAGCAGCAGCA:p.G78fs,ATXN3:NM_001164777:exon3:c.112_113insCAGCAGCAGCAGCAGCAGCAGCAGCAGCAGCAGCAGCAGCAGCAGCAGCA:p.G38fs,ATXN3:NM_001164776:exon4:c.277_278insCAGCAGCAGCAGCAGCAGCAGCAGCAGCAGCAGCAGCAGCAGCAGCAGCA:p.G93fs,ATXN3:NM_001164778:exon6:c.430_431insCAGCAGCAGCAGCAGCAGCAGCAGCAGCAGCAGCAGCAGCAGCAGCAGCA:p.G144fs,ATXN3:NM_001164779:exon6:c.552_553insCAGCAGCAGCAGCAGCAGCAGCAGCAGCAGCAGCAGCAGCAGCAGCAGCA:p.G185fs,ATXN3:NM_001164780:exon7:c.378_379insCAGCAGCAGCAGCAGCAGCAGCAGCAGCAGCAGCAGCAGCAGCAGCAGCA:p.G127fs,ATXN3:NM_001127697:exon8:c.762_763insCAGCAGCAGCAGCAGCAGCAGCAGCAGCAGCAGCAGCAGCAGCAGCAGCA:p.G255fs,ATXN3:NM_001164781:exon8:c.705_706insCAGCAGCAGCAGCAGCAGCAGCAGCAGCAGCAGCAGCAGCAGCAGCAGCA:p.G236fs,ATXN3:NM_001127696:exon9:c.870_871insCAGCAGCAGCAGCAGCAGCAGCAGCAGCAGCAGCAGCAGCAGCAGCAGCA:p.G291fs,ATXN3:NM_030660:exon9:c.750_751insCAGCAGCAGCAGCAGCAGCAGCAGCAGCAGCAGCAGCAGCAGCAGCAGCA:p.G251fs,ATXN3:NM_004993:exon10:c.915_916insCAGCAGCAGCAGCAGCAGCAGCAGCAGCAGCAGCAGCAGCAGCAGCAGCA:p.G306fs | |

**Supplementary Figure 1: Sequence electropherogram of *LRRK2* c.4322G>A:p.R1441H.**

Three representative sequences were shown. Healthy sibling A-II-1 has wild type (W/W), patient B-III-2 has heterozygous (W/M), and patient A-II-3 has homozygous (M/M) c.4322G>A variant (arrowhead) in *LRRK2* exon 31.


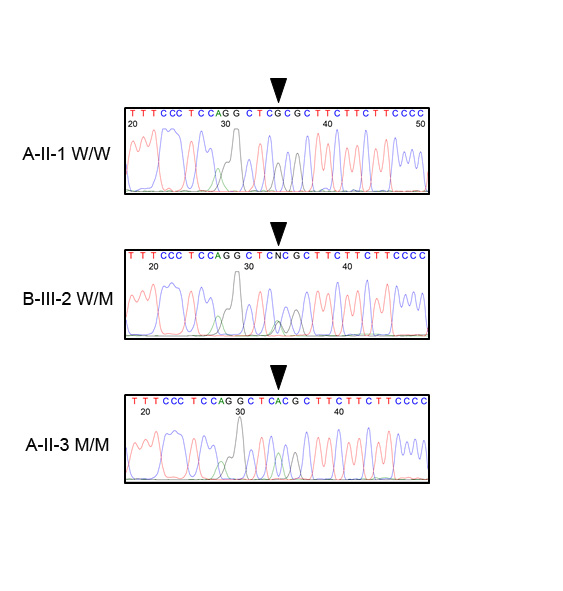


**Supplementary figure 2: Comparison of haplotypes of *LRRK2* region.**

All patients of families A and B harboring *LRRK2* c.4322G>A:p.R1441H sheared common haplotype from D12S2080 to D12S2522 (in bold).


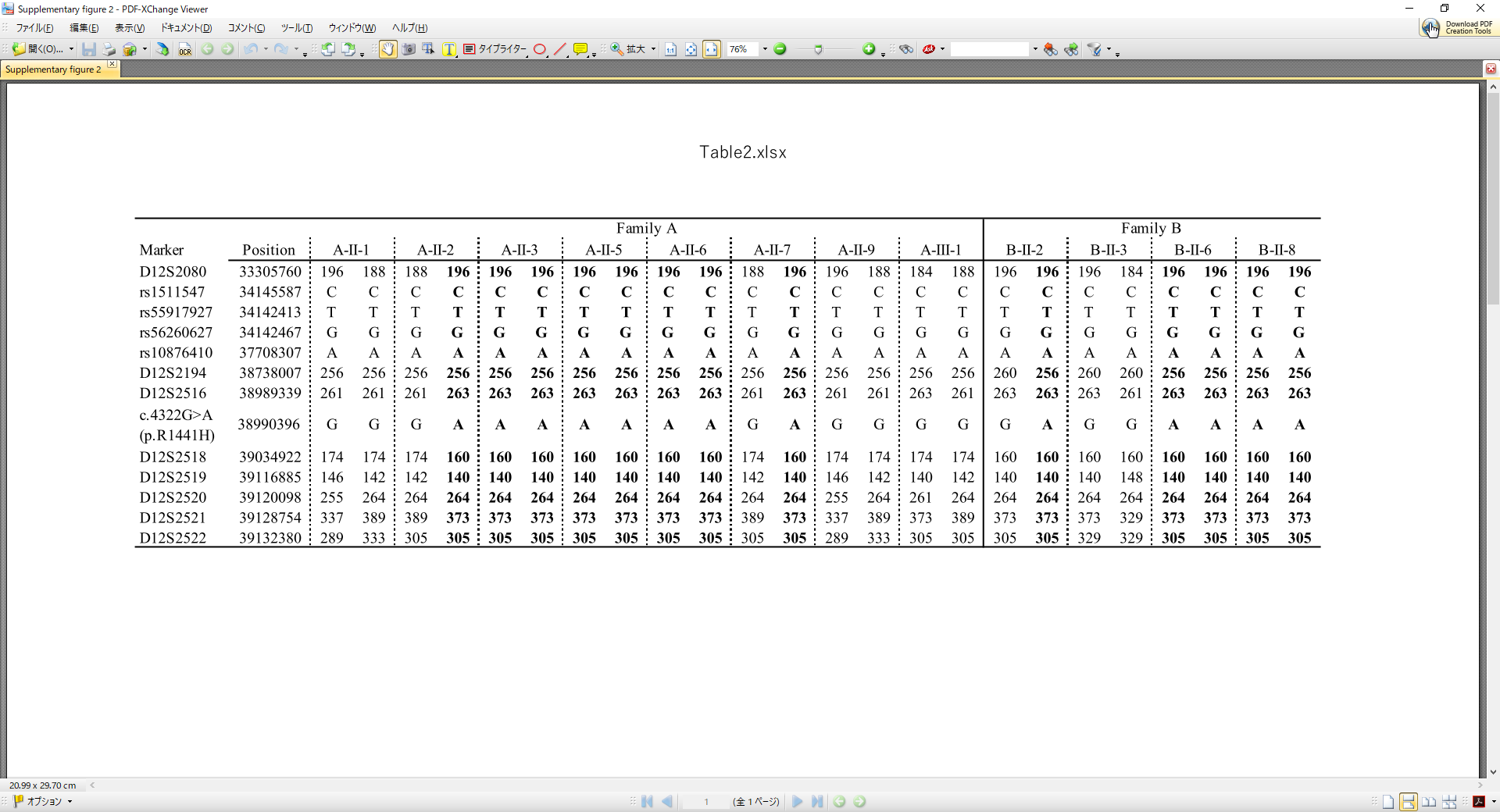

Supplement: Supplementary file 1 — Table S1. Consensus nonsynonymous variants detected by whole genome sequencing. A portion of the output from ANNOVAR is shown. Most of the variants are indel and might be misaligned variant calls. NA: not applicable. Figure S1. Sequence electropherogram of LRRK2 c.4322G > A:p.R1441H. Three representative sequences are shown. Healthy sibling A-II-1 has wild type (W/W), patient B-III-2 has heterozygous (W/M), and patient A-II-3 has homozygous (M/M) c.4322G > A variant (arrowhead) in LRRK2 exon 31. Table S2 Comparison of haplotypes of the LRRK2 region. All patients of families A and B harboring LRRK2 c.4322G > A:p.R1441H shared a common haplotype from D12S2080 to D12S2522 (in bold). (DOCX 365 kb) [file 40478_2018_617_MOESM1_ESM.docx]
